# Rapid Antimicrobial Susceptibility Testing Methods for Blood Cultures and Their Clinical Impact

**DOI:** 10.3389/fmed.2021.635831

**Published:** 2021-03-10

**Authors:** Ritu Banerjee, Romney Humphries

**Affiliations:** ^1^Department of Pediatrics, Vanderbilt University Medical Center, Nashville, TN, United States; ^2^Department of Pathology, Microbiology, and Immunology, Vanderbilt University Medical Center, Nashville, TN, United States

**Keywords:** antimicrobial susceptibility testing, phenotypic, genotypic, sepsis, bloodstream infection

## Abstract

Antimicrobial susceptibility testing (AST) of bacteria isolated in blood cultures is critical for optimal management of patients with sepsis. This review describes new and emerging phenotypic and genotypic AST methods and summarizes the evidence that implementation of these methods can impact clinical outcomes of patients with bloodstream infections.

## Introduction

Sepsis, defined as an infection with dysregulated host response leading to life-threatening organ dysfunction, occurred in nearly 49 million incident cases and accounted for 19% of all deaths worldwide in 2017 (1, 2). The burden of sepsis and its attributable mortality vary greatly by geographic region and patient age (3, 4). In the USA, sepsis is the most common cause of in-hospital death and costs greater than $24 billion annually (1, 5). Mortality rates due to bloodstream infection (BSI) range between 12 and 32% in North America and Europe and are even higher in low- and middle-income countries. Mortality is due in part to increasing rates of antimicrobial-resistant pathogens (5–10). Patients infected with resistant pathogens are more likely to receive ineffective empiric antibiotic therapy, which is associated with poor outcomes, including death (11–14). Conversely, treatment with overly broad antibiotics increases risk of adverse drug events and drives further development of resistance (15, 16).

Sepsis is frequently caused by BSIs. In a retrospective analysis of nearly 3 million adult encounters across 409 US hospitals, a positive blood culture was found in 17% of patients with sepsis (17). In a Swiss prospective population-based study, blood culture-proven pediatric sepsis accounted for 66% of all pediatric hospital admissions for sepsis (18). Knowledge of the antimicrobial susceptibility profile of a blood isolate as soon as possible is often critical for optimal management and outcomes of patients with sepsis, enabling de-escalation or escalation of antibiotics to appropriate definitive therapy (19–22). While standard turnaround time for clinical microbiology laboratories to isolate, identify, and perform antimicrobial susceptibility testing (AST) of bacterial isolates is 48–96 h from the time a blood culture turns positive (23), many rapid testing methods provide results within 6–24 h (24, 25). These novel diagnostics are routinely used in many hospitals; however, the clinical benefit of these methods has not been well-quantified. In this review, we describe currently available rapid AST methods along with the data that support their clinical benefit to patients with sepsis and bacteremia. Fungemia is not discussed, as to date, rapid methods focus on identification of *Candida* in blood cultures, but not susceptibility testing.

## Antimicrobial Susceptibility Testing Methods

US and European regulatory agencies require novel AST methods to generate results that are substantially equivalent to those of the international reference method, broth microdilution (BMD) (26). BMD is fraught with technical limitations that make these correlations challenging, not the least of which include need for a bacterium isolated in pure culture (which may artificially select out a subpopulation of microorganism that grows best *in vitro*), use of culture media that are a poor mimic of the physiological environment of the body, and use of an inoculum size that is infrequently observed in clinical specimens [i.e., 10^8^ colony-forming units (CFU)/ml] (27–30). Despite these limitations, BMD is thought to provide a reasonable, albeit imperfect, correlation to treatment outcomes (31) with decades of data. It is important to recognize that results obtained from new AST methods are “fit” to match those obtained for the reference BMD during test development, which prohibits discovery of outputs that may be better predictors of clinical outcome than the minimum inhibitory concentration (MIC). As such, novel methods for determination of AST have focused primarily on developing a more rapid result—by two primary approaches: those that evaluate a microorganism's phenotype and those that evaluate its genotype (32). In general, rapid phenotypic methods are replacement technology for traditional AST tests used in the clinical laboratory, as these can predict both susceptibility and resistance to an antimicrobial and correlate reasonably well with reference BMD (32). In contrast, full correlation between the genotype and BMD has remained elusive (33–35), and genotypic methods are universally backed up with a phenotypic susceptibility test that is performed upon isolation of the microorganism from blood cultures. A combination of genotypic and phenotypic methods may provide both prediction of susceptibility (if no resistance gene is detected) and AST results for antimicrobials with multifactorial and unclear resistance mechanisms (for example, daptomycin) (32, 36–40).

## Novel Phenotypic Methods for Rapid Antimicrobial Susceptibility Testing From Positive Blood Cultures

Historically, off-label use of positive blood culture broth as the inoculum for disk diffusion or automated AST methods (e.g., Vitek 2) was widely performed in clinical laboratories to expedite time to results for AST from positive blood cultures (41). This was possible, as most blood cultures are monomicrobial and the concentration of bacteria in a positive blood culture approximates a 0.5 McFarland, the inoculum concentration used for traditional AST tests (42). However, the practice became less widespread in the USA following implementation of more stringent laboratory regulation in the form of the Clinical Laboratory Improvement Amendments, which placed stricter controls over laboratory-developed tests. To help with this dilemma, standardization of a direct-from-blood culture disk diffusion method has been undertaken by two laboratory standards groups: the European Committee on Antimicrobial Susceptibility Testing (EUCAST) and the Clinical and Laboratory Standards Institute (CLSI). The EUCAST rapid AST method includes use of positive blood culture as the inoculum for a standard disk diffusion test, which is incubated for 4–8 or 16–18 h. Alternative interpretive criteria are provided for some organisms/antimicrobials at 4–8 h of incubation, which include a “area of technical uncertainty,” i.e., an indeterminate result for some antimicrobials and organisms (43). CLSI's method is similar to that of EUCAST, with plans to publish in 2021 (Audrey Schuetz, personal communication to RMH). While more rapid, these methods remain manual and labor-intensive. A large international evaluation of the EUCAST method demonstrated that 88% of results could be read and 70% interpreted at the 4-h timepoint, which improved to 99 and 85%, respectively, by 6 h (44). Total laboratory automation (TLA) instrumentation may allow for automated setup and reading of disk diffusion zones, providing increased consistency and throughput, although application of this method has not been widely done (45).

More sophisticated approaches to rapid AST from blood cultures use alternative approaches to evaluate the phenotype by applying approaches such as microscopic evaluation of antimicrobial-induced changes to cell morphology, evaluation of division rates, or gene expression (37) (Table 1). Two rapid AST methods are currently approved by regulatory agencies and clinically in use for rapid phenotypic AST from positive blood cultures: the Accelerate PhenoTest BC^®^ (Tucson, AZ), which is both US Food and Drug Administration (FDA) cleared and Conformite-Europeenne *in vitro* Diagnostic (CE-IVD) approved, and the Alfred 60AST (Alifax, Italy), which is CE-IVD. The PhenoTest BC performs rapid identification (ID) (1.5 h) and AST (~7 h) from positive blood cultures by performing fluorescence *in situ* hybridization and time-lapse imaging of bacteria under dark-field microscopy, respectively (39, 46). A variety of morphological and kinetic changes in the bacteria compared with no-antimicrobial controls are used to determine MICs. The PhenoTest has been widely evaluated in the literature and has rapid turnaround time and good performance in US and European studies (46, 49–54). In contrast, the Alfred 60AST utilizes light scattering to detect bacterial growth in a liquid-based culture broth, determining results within 3–5 h. Organism identification is not performed with this latter method and must be determined using alternative methods (47).

**Table 1:** Select rapid phenotypic AST methods that are approved for testing positive blood cultures.

**Test**	**AST technology**	**TTR**	**Regulatory status**	**References**
PhenoTest BC (Accelerate Diagnostics)	Time-lapse imaging of bacterial cells under dark-field microscopy. Morphological and kinetic changes analyzed.	7 h	US FDA cleared, CE-IVD	(46)
Alfred (AliFAX)	Light scattering to detect bacterial growth in liquid culture broth.	3–5 h	CE-IVD	(47)
dRAST (QuantaMatrix)	Time-lapse imaging of bacterial cells on micropatterned plastic microchips.	6 h	CE-IVD	(48)
Reveal AST (Specific Diagnostics)	Sensor array for volatile organic compounds emitted during microorganism growth.	4.5 h	CE-IVD	^1^
ASTar (Q-linea)	Time-lapse imaging of bacterial growth in broth.	3–6 h	CE-IVD	^2^
Fastinov	Flow cytometry applying fluorescent dyes that reveal cell damage during treatment.	80 min	CE-IVD	^3^
LifeScale (Affinity Biosensors)	Mass measurement using a microcantilever.	4 h	CE-IVD	^4^

*AST, antimicrobial susceptibility testing; TTR, time to result*.

1 *https://specificdx.com/reveal-ast (accessed November 30, 2020)*.

2 *https://www.qlinea.com/our-products/astar/astar-instrument/ (accessed November 30, 2020)*.

3 *http://www.fastinov.com/ (accessed November 30, 2020)*.

4 *http://www.lifescaleinstruments.com/Products/Clinical (accessed November 30, 2020)*.

In addition to these methods, a number of novel technologies are in late-phase development, which seeks to increase the speed of phenotypic testing. These technologies evaluate morphological and/or physiological responses earlier in the course of antimicrobial exposure than the traditional 16–20 h. Examples of responses include changes to cell size, mass, membrane integrity, metabolism, and DNA transcription; these approaches are reviewed in detail elsewhere (37, 38). Among the many methods in development, several have achieved CE-IVD, although are not yet in distribution. Among these, the dRAST (QuantaMatrix, Inc., Seoul, Republic of Korea) (55) and ASTar^®^ (Q-linea, Sweden) (56) methods both evaluate morphological changes by time-lapsed microscopic imaging of bacterial cells exposed to antimicrobials. Both yields results in ~6 h and requires off-line identification of the bacteria. More novel approaches include those taken by LifeScale (Affinity Biosensors, Santa Barbara, CA), which measures impact of antimicrobials to bacterial cell mass via a microcantilever (57), Fastinov (Portugal), which evaluates cell by flow cytometry (58), and Reveal™ AST (Specific Diagnostics, Mountain View, CA) (see table footnote 1), which utilizes sensor arrays to measure changes to volatile organic compounds emitted during bacterial growth. The extent to which these methods may correlate with BMD in full-scale clinical trials remains to be determined, but early results are promising (37, 38).

## Genotypic Methods

Genotypic methods in clinical use today are supplemental, not replacement, technology to traditional AST (32). These methods detect the presence/absence of one or more resistance genes, which predict antimicrobial resistance to a single class of antimicrobials. As an example, detection of *mecA* in a blood culture that also harbors *Staphylococcus aureus* predicts methicillin resistance, but additional testing is required to confirm susceptibility to other antimicrobial agents, such as vancomycin, daptomycin, or linezolid. Furthermore, it is rare for complete correlation between genotype and phenotype to be observed—key examples are the presence/absence of *mecA* or *vanA/B*, which predicts resistance/susceptibility to oxacillin in staphylococci or vancomycin in enterococci, respectively. Outside these two examples, antimicrobial resistance is almost always multifactorial and ever-evolving, making such determinations challenging, even with whole-genome sequencing (34, 35), which is poorly standardized (33). Nonetheless, genotypic methods are of value to determine antimicrobial resistance, allowing a more rapid escalation of therapy, which is particularly valuable for patients with sepsis.

Many assays based on the detection of one or more antimicrobial resistance genes present in a positive blood culture are available commercially and routinely used in the clinical laboratory. Most of these are based on polymerase chain reaction (PCR) (Table 2). Only a limited number of genes and their variants are typically queried (i.e., *mecA*/*mecC* for staphylococci, *vanA*/*vanB* for enterococci, and *bla*_*CTX*__−M_ and carbapenemase genes for Gram-negative bacteria). Genetic tests cannot assign a detected resistance gene to a specific bacterium in a polymicrobial specimen, which may result in overcalling resistance. For example, *mecA* detection in a specimen harboring both *S. aureus* and *Staphylococcus epidermidis* could lead to incorrect assigning of this gene to the pathogen, and not the contaminant. Alternatively, rapid detection of a resistance gene in a culture containing multiple Enterobacterales species with differing susceptibilities may reduce undercalling resistance. Furthermore, new mutations and resistance mechanisms are continually evolving, which may limit the ability of certain genetic tests to predict resistance, particularly if a mutation occurs in primer complementary regions (66) or involves overexpression of a gene, like *AmpC*-associated inducible resistance. It is important to note that genotypic methods, in addition to detecting resistance genes, also provide organism identification, and knowledge of select genus or species alone can sometimes guide antimicrobial therapy, particularly for Gram-positive organisms. For example, detection of *Streptococcus pyogenes* should prompt treatment with penicillin. Organism identification without AST is less useful for treatment of Gram-negative organisms, which have diverse and complex resistance mechanisms.

**Table 2:** Select genotypic tests that are approved for rapid detection of resistance markers in positive blood cultures.

**Test**	**Organisms identified**	**Resistance genes**	**References**
Biofire BCID2 (Biofire, Salt Lake City, UT)	9 Gram-positive bacterial targets 14 Gram-negative bacterial targets 7 yeast targets	Carbapenemases *bla_*IMP*_* *bla_*KPC*_* *bla_*OXA*−48−*like*_* *bla_*NDM*_* *bla_*VIM*_* Colistin resistance *mcr-1* ESBL *bla_*CTX*−*M*_* Methicillin *mecA/C* MREJ Vancomycin *vanA/B*	(59)
Verigene BC-GN (Luminex, Austin, TX)	9 Gram-negative bacterial targets	Carbapenemases *bla_*IMP*_* *bla_*KPC*_* *bla_*OXA*−48−*like*_* *bla_*NDM*_* *bla_*VIM*_* ESBL *bla_*CTX*−*M*_*	(60)
Verigene BC-GP (Luminex)	13 Gram-positive bacterial targets	Methicillin *mecA* MREJ Vancomycin *vanA/B*	(61)
ePlex^®^ BCID-GP (GenMark, Carlsbad, CA)	20 Gram-positive bacterial targets “pan” Gram-negative target “pan” Candida target	Methicillin *mecA* MREJ Vancomycin *vanA/B*	(62)
ePlex^®^ BCID-GN (GenMark)	21 Gram-negative targets “pan” Gram-positive target “pan” Candida target	Carbapenemases *bla_*IMP*_* *bla_*KPC*_* *bla_*OXA*−48/*OXA*−23_* *bla_*NDM*_* *bla_*VIM*_* ESBL *bla_*CTX*−*M*_*	(63)
ePlex^®^ BCID-FP (GenMark)	15 Fungal targets	None	(64)
Xpert^®^ MRSA/SA BC (Cepheid, Sunnydale CA)	1 Gram-positive target	Methicillin *mecA*	(65)

## Clinical Impact of Rapid Antimicrobial Susceptibility Testing Methods

The impact of rapid blood culture diagnostic methods on clinical and economic outcomes has been mixed. The majority of outcomes studies to date are single-center, observational studies that evaluate the impact of rapid organism identification with or without AST. Some of these studies have demonstrated decreased time to appropriate antibiotics, lower mortality, shorter durations of hospital and intensive care unit (ICU) stay, and reduced costs, although these are not consistent findings. These studies have been previously summarized elsewhere and have been incorporated in meta-analyses (67–69). Limitations of these studies include their varying quality; small sample sizes; single-center, retrospective, and observational designs; and historical or absent control groups (70–73).

A few studies have used the more rigorous study design of a prospective randomized controlled trial (RCT) to evaluate outcomes of rapid blood culture AST methods and are summarized in Table 3. In general, these RCTs have shown less dramatic benefits than observational studies. The first RCT evaluating the impact of rapid AST on clinical outcomes was published in 1994 and was a single-center trial using the Baxter MicroScan WalkAway-96 system with provision of AST results on either the same day using blood culture broth to inoculate the test (intervention) or the day after using isolated colonies to inoculate the test (control) (74). Patients in the same-day AST group had significantly lower mortality, fewer ancillary tests, and lower health-care costs than those in the control group (74). Unfortunately, more recent studies using newer technologies have not shown such dramatic mortality benefits. A single-center RCT conducted in the Netherlands evaluated the impact of a laboratory-developed semi-molecular AST method combining culture in the presence of antibiotics plus real-time 16S rRNA PCR and found that the rapid test resulted in no appreciable benefits, in terms of either antimicrobial utilization or clinical outcomes, as compared with conventional testing (75). However, in this study, clinicians did not appear to act upon the rapid AST results, perhaps explaining the lack of difference between the study arms. A single-center, randomized, controlled trial evaluated the impact of the FilmArray Blood Culture Identification (BCID) test, which can detect species of bacteria and *Candida* as well as select resistance markers (*mecA, vanA, vanB*, and *bla*_*KPC*_) using multiplex PCR. In this study, participants with positive blood cultures were randomized in the clinical laboratory to have blood culture characterization with either conventional testing methods including matrix-assisted laser desorption–ionization time of flight (MALDI-TOF), the BCID test, or the BCID test plus antimicrobial stewardship review. Participants in both BCID arms had faster time to antibiotic escalation, less treatment of contaminants, and less use of broad-spectrum antibiotics than had those receiving conventional testing. In addition, participants who received BCID paired with antimicrobial stewardship had more rapid antibiotic de-escalation than those who received BCID without stewardship. However, no differences were observed between the arms in mortality, length of hospital stay, infection with multidrug-resistant organisms or *Clostridium difficile*, or cost of care (20). Notably, the BCID test used in this study had greater impact on management of Gram-positive than Gram-negative infections, most likely because the diagnostic enabled detection of only a single resistance determinant (*bla*_*KPC*_) from Gram-negative species, which was exceedingly rare at the time and did not provide rapid phenotypic susceptibility information for a full panel of antibiotics (20).

**Table 3:** Randomized controlled trials evaluating clinical impact of rapid blood culture antimicrobial susceptibility testing methods.

**Study, year, location**	**Study design**	**Rapid (intervention) method**	**SOC (control) method**	**Rapid test performance**	**Outcomes of rapid test compared with standard methods**	**Rapid testing paired with antimicrobial stewardship**	**Comment**
Doern, 1994, USA (74)	Single-center prospective 2-arm RCT (*N* = 573)	Baxter MicroScan WalkAway-96 reported same day	Baxter MicroScan WalkAway-96 reported following day	Time to AST result 16 h faster than SOC	Decreased mortality, ancillary tests, cost Change in antibiotic therapy was 15 h faster in rapid AST arm No difference in LOS	No	Randomization scheme based on first letter of patient last name
Beuving, 2015, Netherlands (75)	Single-center prospective 2-arm RCT (*N* = 250)	Growth in presence of antibiotics assessed by 16S rRNA PCR	BD Phoenix	94% agreement with SOC AST Time to AST result 15 h faster than SOC	Decreased TOT No differences in mortality, LOS	No	Rapid AST was not implemented optimally and results were not used by clinicians Underpowered to detect differences in clinical outcomes
Banerjee, 2015, USA (20)	Single-center, prospective 3-arm RCT (*N* = 617)	BioFire BCID and BCID plus stewardship	MALD-TOF, agar dilution	97% agreement for on-panel organisms 19% of isolated organisms not on rapid test panel Time to AST result 49 h faster than SOC	Decreased TOT Faster time to escalation and de-escalation, less treatment of contaminants, less broad-spectrum antibiotic treatment No differences in mortality, LOS, adverse events, cost	Yes Audit and feedback by ID pharmacist or physician 24/7 in one intervention arm; treatment guidance comments included in microbiology result report for both intervention arms	More impact among Gram-positive than Gram-negative infections Population had low resistance rates Underpowered to detect differences in clinical outcomes
Kim, 2020, Korea (76)	Single-center, prospective 2-arm RCT of patients with hematologic malignancies (*N* = 89)	QMAC-dRAST (QuantaMatrix, Inc.)	MALDI-TOF, MicroScan, VITEK 2	Agreement with SOC not reported. Time to AST result 35 h faster than SOC	Decreased TOT Less broad-spectrum antibiotic use No differences in mortality, *Clostridium difficile*, multidrug-resistant infections	Yes ID team reviewed all patients	Excluded patients in both arms with off-panel organisms
Banerjee, 2020, USA (19)	Multi-center prospective 2-arm RCT of patients with Gram-negative bacteremia (*N* = 448)	Accelerate Pheno System	MALDI-TOF, broth microdilution or agar dilution	Time to AST 36 h faster than SOC	Decreased TOT No differences in mortality, LOS, adverse events, cost	Yes Audit and feedback by ID pharmacist or physician Mon–Friday during the day	Greater impact for more resistant isolates Population had low resistance rates Underpowered to detect differences in clinical outcomes

*PCR, polymerase chain reaction; SOC, standard of care; MALDI-TOF, matrix-assisted laser desorption–ionization time of flight; TOT, time to optimal therapy; LOS, length of stay; AST, antibiotic susceptibility testing; ID, infectious diseases; BCID, Blood Culture Identification*.

The recently completed RAPIDS GN trial evaluated the impact of a rapid phenotypic AST method for Gram-negative bacilli bloodstream infection and addresses some of the limitations of the BCID trial (19). This multicenter study evaluated the impact of the Accelerate PhenoTest™ BC Kit, performed on the original FDA-cleared Accelerate Pheno™ System, compared with standard of care (SOC) MALDI-TOF and BMD or agar dilution for AST. In this study, all blood cultures were reviewed by the stewardship clinicians. The arm with rapid testing had faster time to antibiotic change and optimal antibiotic therapy but did not have any benefit in terms of mortality, length of stay, adverse events, or cost. Notably, the impact on antibiotic utilization varied by resistance profile of the blood isolate; compared with the SOC arm, in the rapid testing arm, time to any Gram-negative antibiotic change occurred 24 h faster for all patients, and antibiotic escalation occurred 43 h faster for patients with drug-resistant isolates (19). Lastly, a recent small study from Korea evaluated the impact of a rapid phenotypic AST method based on a microscopic imaging and microfluidic chip technology called dRAST (QuantaMatrix) (76). In this study, patients with hematologic malignancies and bacteremia were randomized to receive either the rapid testing method or conventional testing, which consisted of MALDI-TOF and MicroScan (Beckman Coulter Inc., Atlanta, GA) and VITEK 2 systems (bioMérieux, Inc.) for AST. Time to optimal therapy was significantly faster in the rapid testing arm than in the control arm, although there were no differences in mortality or other clinical outcomes (76).

Taken together, these studies demonstrate that a variety of rapid AST methods can shorten time to optimal therapy and improve antibiotic stewardship for patients with bloodstream infections. However, most RCTs do not demonstrate that rapid AST methods result in significant reductions in mortality, hospital length of stay, or adverse events, perhaps because larger studies are required to detect differences in these rare events. It is also notable that with the exception of the study by Kim et al., most of these trials were conducted in areas with low rates of antimicrobial-resistant bacteria, and it is possible that more clinical benefit would be observed in areas with higher rates of resistant infections (19, 20). Additionally, most of the studies demonstrate the importance of pairing rapid blood culture diagnostics with antibiotic stewardship team review, as has been emphasized by many others (20, 68, 69, 73, 77–80). A meta-analysis of primarily observational studies demonstrated mortality benefit when blood culture diagnostics were used with stewardship programs but not without stewardship programs (69). A decision analytic model demonstrated that rapid blood culture diagnostics were more cost-effective if implemented with stewardship than without stewardship (80).

## Conclusion

In conclusion, technological advances that enable rapid AST from organisms growing in blood cultures have great potential to improve the care and outcomes for patients with sepsis. Numerous platforms are currently available or in development, all of which can provide rapid genotypic or phenotypic detection of resistance. However, the success of these technologies requires demonstration of clinical impact. Unlike trials that evaluate a direct action, RCTs conducted for rapid diagnostics are one step removed from the patient, requiring the physician to act on the result. This task has been shown to be best supported when ASPs are active participants in RCTs, showing significant reduction in time to therapy optimization through the use of rapid AST devices. However, the impact of more rapid therapeutic intervention remains largely theoretical. Shorter time to optimal antibiotic therapy should lead to reduced length of hospitalization and mortality, but studies conducted to date were not sufficiently powered to measure these endpoints. More subtle endpoints (e.g., impact to microbiome), alternative trial designs, and inclusion of patient preferences in endpoint determinations may all provide further insight into the value of these tests. Also worth noting is the fact that while significant improvement in hospitalization stays or mortality has not been demonstrated through rapid antimicrobial de-escalation, the opposite is also true—i.e., interventions conducted ~1–2 days earlier in the course of sepsis do no harm. These data provide a valuable foundation to aid improved stewardship of antibiotics.

Another area of much-needed future research is implementation science. While most large academic hospitals have adopted rapid AST methods for blood cultures, their use is not universal (81). Similarly, extending these technologies to specimens other than positive blood cultures is challenging due to the high frequency of specimens that would need to be tested prior to a single positive result. For example, a recent survey of US hospital data for bronchoalveolar lavage specimens demonstrated only a third of specimens yielded a positive result, meaning expensive technology would be performed on two thirds of specimens with no results (82). Identifying patient populations likely to have the most benefit from these methods and determining how to encourage clinicians to act on rapid AST results are both critical for further development of rapid AST devices. Furthermore, determining the relative value of phenotypic vs. genotypic rapid AST methods is needed. To this end, an RCT is underway to evaluate patients with positive blood cultures tested by a genotypic (control) vs. rapid phenotypic (intervention) approach; the primary endpoint is duration of anti-pseudomonal beta-lactam therapy and anti-methicillin-resistant *Staphylococcus aureus* (anti-MRSA) therapy (clinicaltrials.gov, NCT03744728). Ongoing investment in infectious disease diagnostics and development of rapid AST technologies will be important for continued improvements in sepsis outcomes.

## Author Contributions

RB and RH reviewed the literature, drafted, and revised the manuscript. All authors agree to be accountable for the content of the work.

## Conflict of Interest

The authors declare that the research was conducted in the absence of any commercial or financial relationships that could be construed as a potential conflict of interest.
